# Chronic alcohol-induced brain states limit propagation of direct cortical stimulation

**DOI:** 10.1038/s41598-025-21802-z

**Published:** 2025-10-10

**Authors:** Bettina Habelt, Nasibeh Talebi, Dzmitry Afanasenkau, Cindy Schwarz, Beate Knauer, Marcus W. Meinhardt, Rainer Spanagel, Carsten Werner, Ivan R. Minev, Christian Beste, Nadine Bernhardt

**Affiliations:** 1 Department of Psychiatry and Psychotherapy, Faculty of Medicine Carl Gustav Carus, Technische Universität Dresden, Dresden, Germany; 2 Leibniz Institute of Polymer Research Dresden, Dresden, Germany; 3 Cognitive Neurophysiology, Department of Child and Adolescent Psychiatry, Faculty of Medicine, Technische Universität Dresden, Dresden, Germany; 4 Biotechnology Center (BIOTEC), Center for Molecular and Cellular Bioengineering (CMCB), Technische Universität Dresden, Dresden, Germany; 5 Electrophysiology Facility (EcrtD) of the Center for Regenerative Therapies Dresden (CRTD), Technische Universität Dresden, Dresden, Germany; 6 Institute of Psychopharmacology, Central Institute of Mental Health, Medical Faculty Mannheim, University of Heidelberg, Mannheim, Germany; 7 Department of Molecular Neuroimaging, Medical Faculty Mannheim, Central Institute of Mental Health, University of Heidelberg, Heidelberg, Germany; 8 German Center for Mental Health (DZPG), Mannheim/Heidelberg/Ulm, Germany; 9 Else Kröner Fresenius Center for Digital Health, Faculty of Medicine Carl Gustav Carus, Technische Universität Dresden, Dresden, Germany; 10 German Center for Child and Adolescent Health (DZKJ), Leipzig/Dresden, Germany

**Keywords:** Preclinical research, Translational research, Biomarkers, Prognostic markers, Addiction

## Abstract

The impact of chronic alcohol consumption is evident in disruptions within prefrontal circuitries, resulting in cognitive deficits that underlie lost control over drinking. Given the limited efficacy of current pharmacotherapy, we employed multifunctional bioelectronics to investigate the potential of direct electrical brain stimulation to rectify neural impairments in alcohol-dependent rats. Cortical stimulation counteracted the observed deficiencies in event-related brain potentials and neural oscillations following chronic alcohol consumption, particularly by strengthening low-beta oscillations related to executive control. Stimulation efficacy thereby correlated with individual drinking patterns. In alcohol-dependent rats, stimulation-related neuroenhancement was spatially confined to the stimulation site, indicating reduced neural connectivity, as confirmed by a computational approach to directed functional connectivity patterns. Such state-dependent factors may contribute to the varying efficacy of brain stimulation observed in patients, ultimately suggesting the need for personalized neuromodulation designs.

**Supplementary Information:**

The online version contains supplementary material available at 10.1038/s41598-025-21802-z.

## Introduction

Alcohol is the most commonly abused drug worldwide. An estimated 400 million individuals live with alcohol use disorders (AUD), which constitute a substantial part of the overall burden of disease and widespread social harm^1^. Despite the availability of pharmacological and psychotherapeutic interventions, a significant portion of cases show treatment resistance and high relapse rates^2,3^.

Deficiencies in behavioral control manifesting in compulsive drinking and increased relapse probability are a hallmark of AUD^4^ and are related to impairments in the prefrontal cortex (PFC) and altered functional connectivity within frontostriatal circuitries^5–8^. Evidence from both animal models and human studies suggests that electrical neuromodulation targeting these addiction-related neural networks may offer a viable addition to conventional therapy schemes^9–11^. Deep brain stimulation (DBS) via implanted probes and noninvasive techniques such as transcranial direct current stimulation (tDCS) have shown promise in reducing craving and relapse in AUD patients^12,13^. However, the limited understanding of the precise mechanisms and potential factors affecting the current distribution poses a risk of inadequate treatment design and varying therapeutic effectiveness^14–16^.

Within the three-stage heuristic model of addiction (intoxication, withdrawal and preoccupation) that outlines the domains of dysfunction in AUD and the mediating neurobiology^6^, any stage can engage neuroadaptations that lead to compulsive-like alcohol consumption^17^. Specifically, chronic alcohol use and acute withdrawal generate prominent changes at synapses, including compensatory effects on the expression, localization, and function of synaptic proteins, channels, and receptors, as well as changes in signal transduction that trigger longer-term molecular, cellular, and network adaptations^18–20^. Such neuroadaptations become evident in electrophysiological recordings of brain function^21–24^. As a result, neural activity measures such as event-related brain potentials and neural oscillations have been recognized as valuable biomarkers to aid in AUD prevention, diagnosis, therapy and rehabilitation^25–28^, providing objective indicators of treatment efficacy and relapse risk^29–31^. Moreover, incorporating these biomarkers into brain stimulation approaches would increase precision, enabling personalized neuromodulation with stimulation parameters dynamically adjusted to individual neural activity patterns. Nevertheless, research exploring how brain stimulation modulates neurophysiological signatures related to AUD is rare^32,33^.

Thus, in this preclinical animal study, we sought to investigate the efficacy of electrical neuromodulation in reversing long-term alcohol-induced alterations in neural activity. For this purpose, we utilized a bidirectional electrocorticographic (ECoG) interface, which was implanted on the surface of the medial PFC, to circumvent current attenuation through the skull vie transcranial approaches for more effective and precise recording and stimulation close to the target structure while remaining less invasive than DBS^34,35^. Long-term alcohol consumption with high translational value was modeled in rats, using the alcohol deprivation effect (ADE) protocol. This well-established model reflects addiction-like alcohol consumption comparable to the human condition and provides excellent face validity to the relapse behavior reported in patients according to the Diagnostic and Statistical Manual of Mental Disorders^36,37^. The ADE rat model has been utilized in various preclinical and translational alcohol studies to identify new treatment targets with good predictive validity^36^. Previously, we showed that the ADE model displays characteristic changes in auditory event-related potentials (ERPs) and event-related oscillations (EROs) attributable to long-term alcohol consumption and associated with an increased relapse probability^38^. As the first significant contribution of the present study, we demonstrate that prefrontocortical stimulation can reverse these long-term alcohol-induced neural signatures. Despite a general rectification of neural activity patterns, our second major finding indicates that the extent and spatial spread of stimulation-related responses compared with those of alcohol-naïve controls remain limited, suggesting a lasting AUD signature that is robust to neuromodulation, which may also underlie the varying effectiveness of brain stimulation in patients.

## Results

**Fig. 1:**
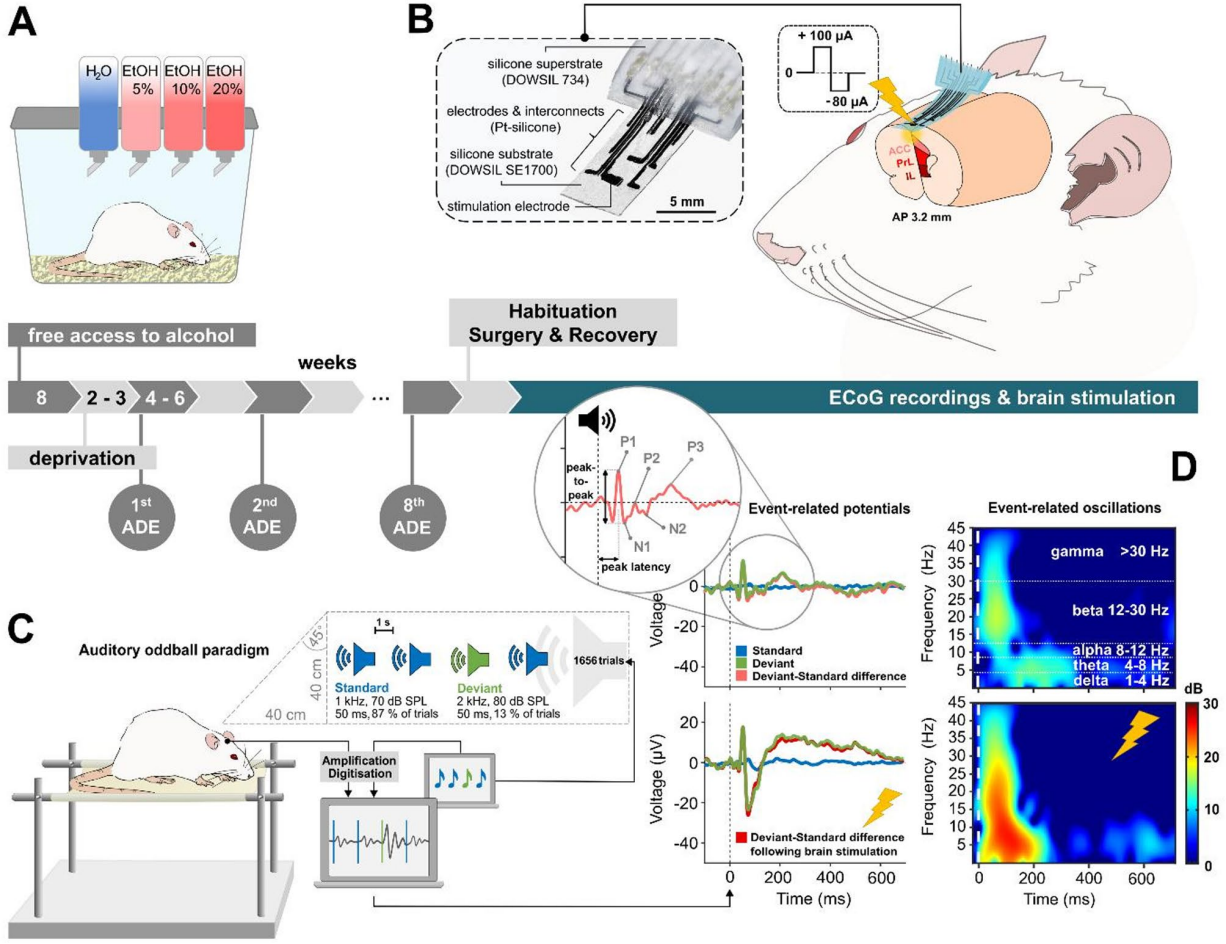
Experimental protocol for long-term (1 year) alcohol consumption and neuromodulation in the ADE rat model. (**A**) Alcohol was freely available for the first 8 weeks, followed by periods of free access and deprivation of variable lengths to prevent habituation. (**B**) Following the final (8th) alcohol drinking cycle, the animals were habituated to the recording setup and underwent stereotactic surgery to implant the neuroprosthetic interface above the medial PFC encompassing anterior cingulate (ACC), prelimbic (PrL) and infralimbic (IL) cortices. The stimulation electrode was located 3.2 mm anterior to bregma. Bilateral stimulation was delivered epidurally as biphasic, charge-imbalanced rectangular pulses (100 µA/-80 µA, 130 Hz, 100 µs pulse width) for 20 min right before recording. (**C**) Animals underwent initial electrocorticographic (ECoG) recordings during a passive two-tone auditory oddball paradigm 2 weeks following the last consumption of alcohol and three days postsurgery, with a subsequent session following stimulation another three days later. (**D**) Exemplary grand average data derived from the frontocentral stimulation electrode site, illustrating deviant-minus-standard difference ERP (left) and ERO (right) (analyzed as event-related spectral perturbation (ERSP) in decibels (dB)) across delta (1–4 Hz), theta (4–8 Hz), alpha (8–12 Hz), beta (12–30 Hz) and gamma (> 30 Hz) frequency ranges before (top) and after (bottom) neuromodulation, highlighting stimulation-induced neuroenhancement. ERP figures further illustrate habituation to frequent standard sounds, as indicated by the flattened neural response, which remains unaffected by stimulation.

Alcohol-dependent rats were obtained through a paradigm of long-term alcohol consumption characterized by alternating phases of voluntary alcohol intake and forced abstinence with variable lengths to prevent behavioral adjustment (Fig. 1A). Following renewed access to alcohol after withdrawal, animals display temporarily increased alcohol consumption, termed the alcohol deprivation effect (ADE). As compulsive alcohol intake, a hallmark of AUD, manifests in the model only after a protracted duration^36^, the paradigm was administered over a 13-month period. Consequently, the animals were of advanced age at the time of electrophysiological recordings. Given that neural activity^39,40^ and susceptibility to the effects of alcohol^41–43^ vary with age, all the animals were age-matched such that the observed changes in neural activity could be attributed specifically to prolonged alcohol exposure and the applied electrical stimulation.

At the end of the ADE protocol and under abstinence conditions, the animals were implanted with ECoG devices (Fig. 1B). Prefrontal ERPs and EROs to acoustic stimuli were acquired minimal 2 weeks following the last alcohol intake, during a passive two-tone auditory oddball paradigm (Fig. 1C, D) prior to and following electrical stimulation of the medial PFC (Supplementary data 1, 2). Direct cortical stimulation via ECoG is a novel treatment approach for AUD, but its duration of effect remains unknown. Therefore, to ensure that the baseline recordings were not influenced by any residual stimulation effects, test order was not counterbalanced and all the animals were first recorded without stimulation. The applied electrical stimulation protocol uses biphasic, charge-imbalanced pulses, which decrease the resting membrane potential and facilitate neural excitability^44,45^ while simultaneously reducing adverse tissue reactions and dissolution of electrode materials^46,47^. Positively charged (anodal) stimulation was used, as it proved to be more effective than negatively charged (cathodal) stimulation, as it requires lower stimulation intensities, excites more neurons and induces higher spiking responses^48–51^. To prevent habituation between the two sessions of the auditory oddball paradigm, we implemented a three-day interval between sessions and presented a high number of stimuli (> 1600) in a randomized and unique order for each session. Within each session, as expected and consistent with our previous investigations in alcohol-naïve animals^52^, rats presented attenuated neural responses to frequent standard sounds, indicating habituation due to high repetition^53^ and sustained responses to deviants. Given the relevance of deviant-minus-standard responses for assessing attentional processing, working memory, and inhibitory control^54^ as key features underlying AUD symptomatology, subsequent analysis focused on this differential measure.

Three-factor repeated measures (rm) analysis of peak latencies of ERPs P1, N1, P2, N2 and P3, peak-to-peak ERP amplitudes (P1N1, N1P2, P2N2, N2P3) and bandpower (event-related spectral perturbation (ERSP)) as well as latency and frequency with maximum power of EROs within delta, theta, alpha, beta and gamma bands from alcohol-dependent animals (n = 10) and naïve controls (n = 10), revealed, that both, alcohol and stimulation, induced significant changes in neural activity (group effect:* F*(27, 216) = 1218.940, *p* < 0.001; treatment effect (*F*(27, 216) = 919.359,* p* < 0.001, Supplementary data 3). Further analyses, which were conducted separately for each of the electrophysiological parameters, also indicate an influence of electrode location (rmANOVA; Supplementary data 4, 5, 6).

### Prefrontal neural activity prior to electrical neuromodulation

**Fig. 2:**
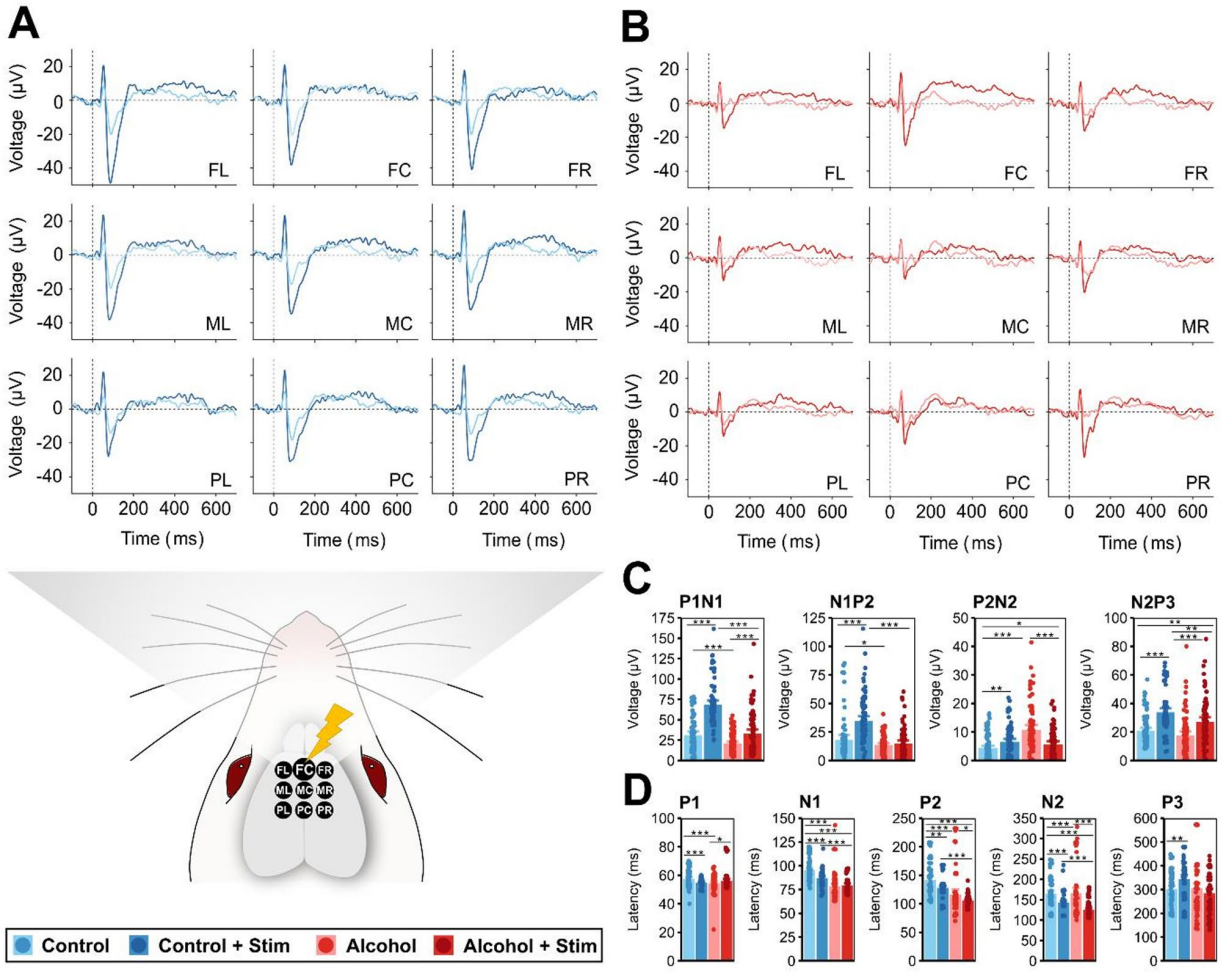
Impact of alcohol consumption and prefrontocortical electrical stimulation on auditory event-related potentials in the ADE rat model. Grand average deviant-minus-standard ERP difference curves of (**A**) controls and (**B**) alcohol-dependent animals at all electrode sites, labeled according to their position above the medial PFC as frontocentral (FC), frontal left (FL), frontal right (FR), medial central (MC), medial left (ML), medial right (MR), posterior central (PC), posterior left (PL) and posterior right (PR). (**C**) Peak-to-peak amplitudes and (**D**) latencies of deviant-minus-standard ERP differences. Panels **C-D** display data points for all channels of all the rats and mean barplots ± 95% confidence intervals. Data for naïve controls (blue) and alcohol-dependent animals (red) prior to stimulation are presented in light colors and following stimulation in dark colors. Asterisks indicate significant differences at *p < 0.05, **p < 0.01, and ***p < 0.001.

**Fig. 3:**
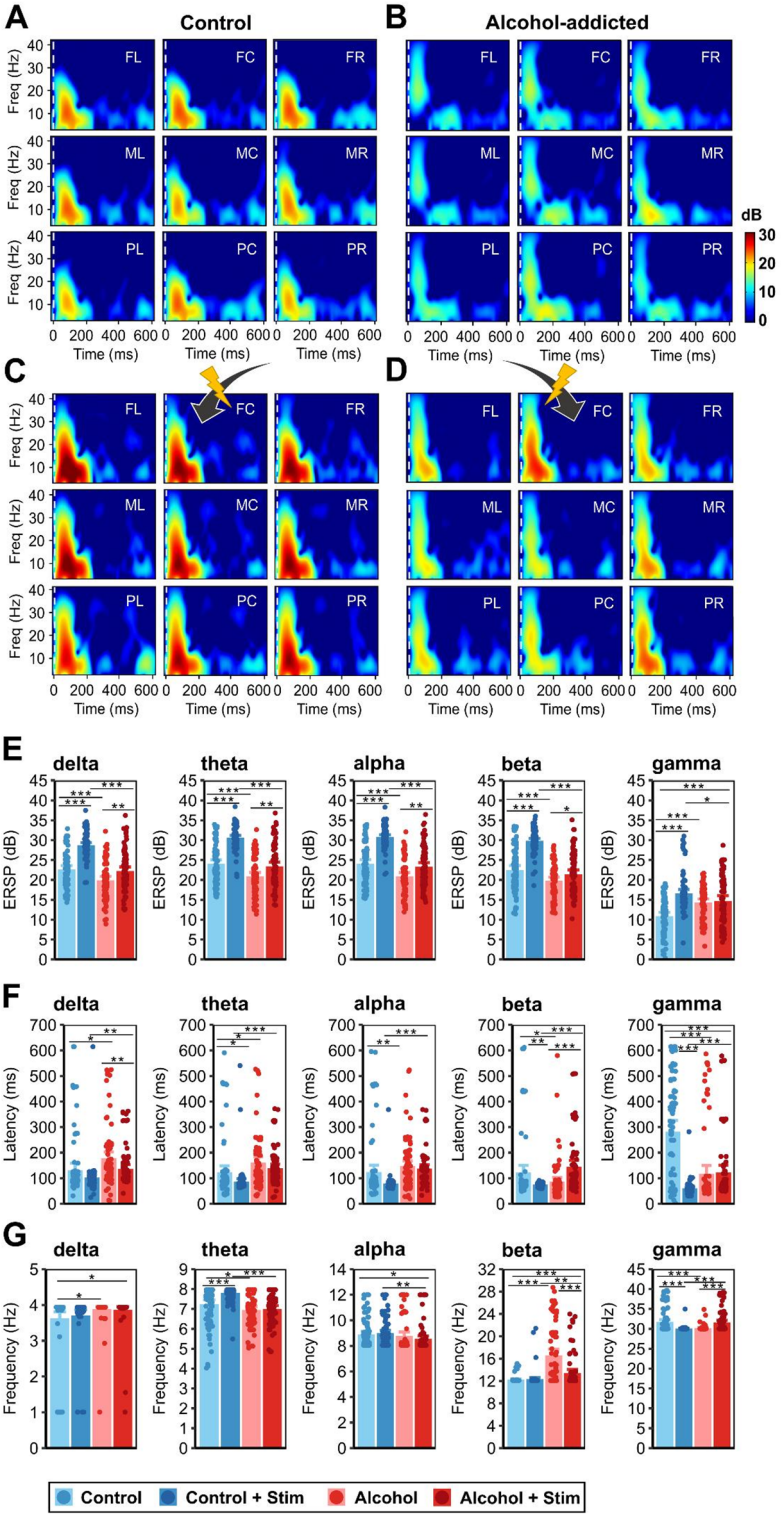
Impact of long-term alcohol consumption and direct cortical stimulation on prefrontal auditory event-related brain dynamics in the ADE rat model. Grand average deviant-minus-standard ERSPs (in dB) in the frequency range of 1–45 Hz in (**A**) naïve controls and (**B**) alcohol-dependent animals prior to and following prefrontocortical stimulation (**C**, **D**). (**E**) Maximum ERO activity, (**F**) Latencies and (**G**) Frequencies of maximum ERO activity within the delta (1–4 Hz), theta (4–8 Hz), alpha (8–12 Hz), beta (12–30 Hz) and gamma (> 30 Hz) frequency bands and over the whole frequency range. Panels E–G display data points for all channels of all the rats and mean barplots ± 95% confidence intervals. Data for naïve controls (blue) and alcohol-dependent animals (red) are presented in light colors prior to stimulation and in dark colors following stimulation. Asterisks indicate significant differences at *p < 0.05, **p < 0.01, and ***p < 0.001.

Prior to stimulation, alcohol-dependent animals presented reduced P1N1 and N1P2 components (Fig. 2A–C; Supplementary Data 7), resulting in earlier peaks of N1 and P2 (Fig. 2D; Supplementary Data 7). In contrast, the P2N2 amplitude increased following long-term alcohol consumption. Corresponding oscillatory activity (ERSP) across delta (1–4 Hz), theta (4–8 Hz), alpha (8–12 Hz) and beta (12–30 Hz) frequency ranges, exhibited reduced and later peaking amplitudes in alcohol-dependent animals compared with controls (Fig. 3A, B, E, F; Supplementary data 7). Only in the gamma band (30–45 Hz) did long-term alcohol consumption induce an increased oscillatory power. In addition, we observed elevated activity within the beta frequency range in alcohol-dependent animals (Fig. 3B, G; Supplementary data 7). In both groups, the recorded activity was similar across channels (Figs. 3A, B; Supplementary data 8, 9).

### Effects of direct cortical stimulation on prefrontal neural activity

In controls and alcohol-dependent animals, stimulation of the medial PFC (100 µA/-80 µA, 130 Hz, 100 µs pulse width, 20 min) had a marked neuroenhancing effect. However, a significant group × treatment interaction (*F*(27, 216) = 468.095, *p* < 0.001; Supplementary data 3) indicated a different outcome between the groups. In healthy rats, stimulation (*F*(26, 208) = 1176.962, *p* < 0.001, Supplementary data 3) induced a substantial increase in P1N1, N1P2, and N2P3 amplitudes and oscillatory power across the entire frequency spectrum, with earlier peak activity, particularly in the gamma range (Fig. 2A-C, 3C,E, F; Supplementary data 5, 10, 11). Similarly, in alcohol-dependent animals, we observed increased ERP amplitudes, particularly of the P1N1 component, and increasedoscillatory powers following stimulation (Figs. 2A, B 3D, E; Supplementary data 12, 13). Stimulation amplified activity particularly in the low-beta frequency range (Fig. 3G; Supplementary data 12).

Notably, stimulation-induced effects were weaker in alcohol-dependent animals than in controls (Supplementary data 14, 15). They exhibited variation across channel locations, with the most pronounced effects observed at the frontocentral (FC) stimulation electrode site (Figs. 2B, 3D, Supplementary data 16), whereas the controls showed an even distribution across channels (Fig. 3C, Supplementary data 17).

But to what extent has electrical stimulation been able to reinstate healthy-like neural activity in alcohol-dependent subjects? Our findings indicate that in alcohol-dependent animals, stimulation restored ERP amplitudes and low-frequency oscillatory activity to levels comparable to those of healthy nonstimulated controls, whereas elevated high-frequency oscillatory activity persisted (Fig. 2A, B; Supplementary data 18, 19, 20). The reduced latencies of the N1, P2, and N2 components in alcohol-dependent rats (Fig. 3F; Supplementary data 18, 19) may indicate that electrical neuromodulation not only facilitates neural processing but also enhances it beyond normal levels.

### Chronic alcohol-related alterations in prefrontal connectivity affect the current spread of direct cortical stimulation

The variation in event-related neural activity following stimulation observed across channels in alcohol-dependent animals prompted us to pursue additional analyses providing insights into prefrontal connectivity as a critical factor for the effective transmission of electrical signals.

**Fig. 4:**
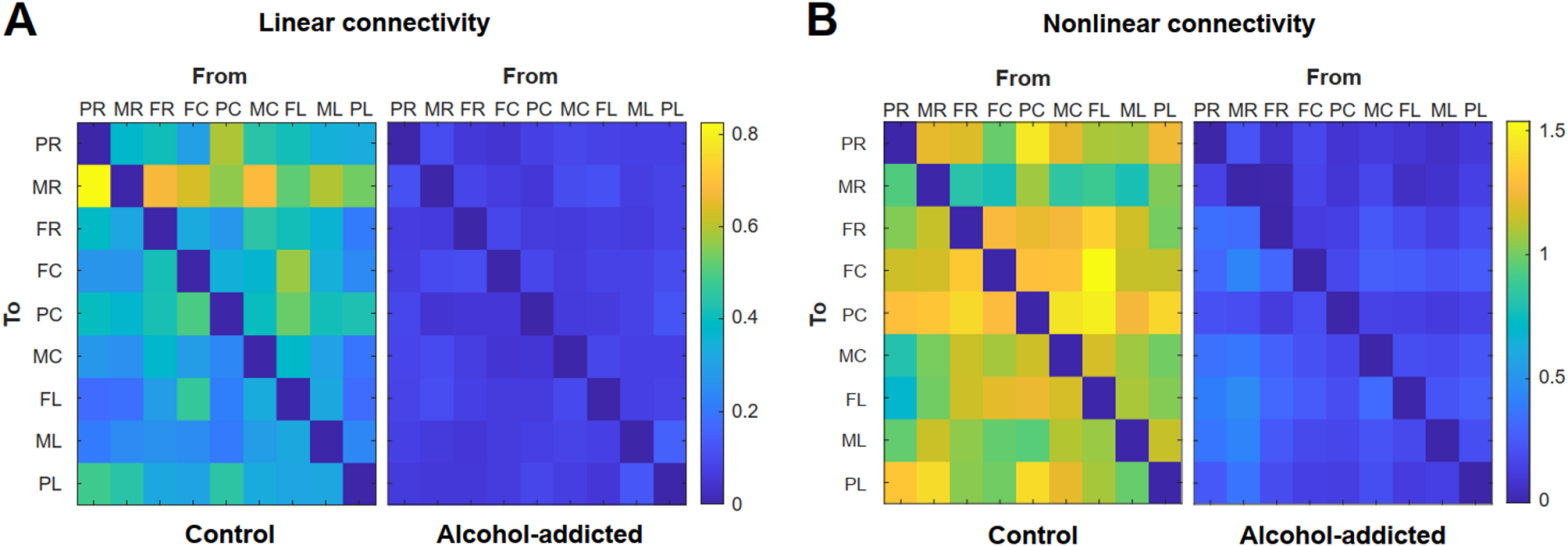
Prefrontal auditory event-related functional connectivity following direct cortical stimulation. Average connectivity patterns between channels of healthy controls (left panels) and alcohol-dependent animals (right panels) for (**A**) linear connectivity and (**B**) non-linear connectivity. ${lC}_{i\to j}$ and ${NC}_{i\to j}$ are shown by the element from electrode *i* to electrode *j*. The self-connections (${lC}_{i\to i}, {NC}_{i\to i}$) have been discarded from the plot representation.

An artificial neural network, the nCREANN approach, was used to reveal linear and non-linear connectivity between channel locations. Compared with those of the controls, the connectivity values of the alcohol-dependent rats were lower after stimulation (Fig. 4), confirming weaker interactions between brain regions. Comparisons across the entire frequency range revealed significantly reduced connectivity between multiple channels in alcohol-dependent animals, particularly for linear connectivity patterns, with mostly moderate to strong effect sizes (Supplementary data 21). Considering the importance of beta oscillatory activity emphasized by our findings, we also compared connectivity results between groups specifically for the low (12–15 Hz), middle (15–18 Hz), and high-beta (18–30 Hz) subranges. We identified sporadic significant differences, particularly in linear connectivity within the high-beta range and non-linear connectivity within mid-beta frequencies (Supplementary data 22). Effect sizes were highly variable, with linear connectivity showing generally higher values, especially within the low-beta range (Supplementary data 22).

### Effects of individual alcohol consumption on the efficacy of direct cortical stimulation

**Fig. 5:**
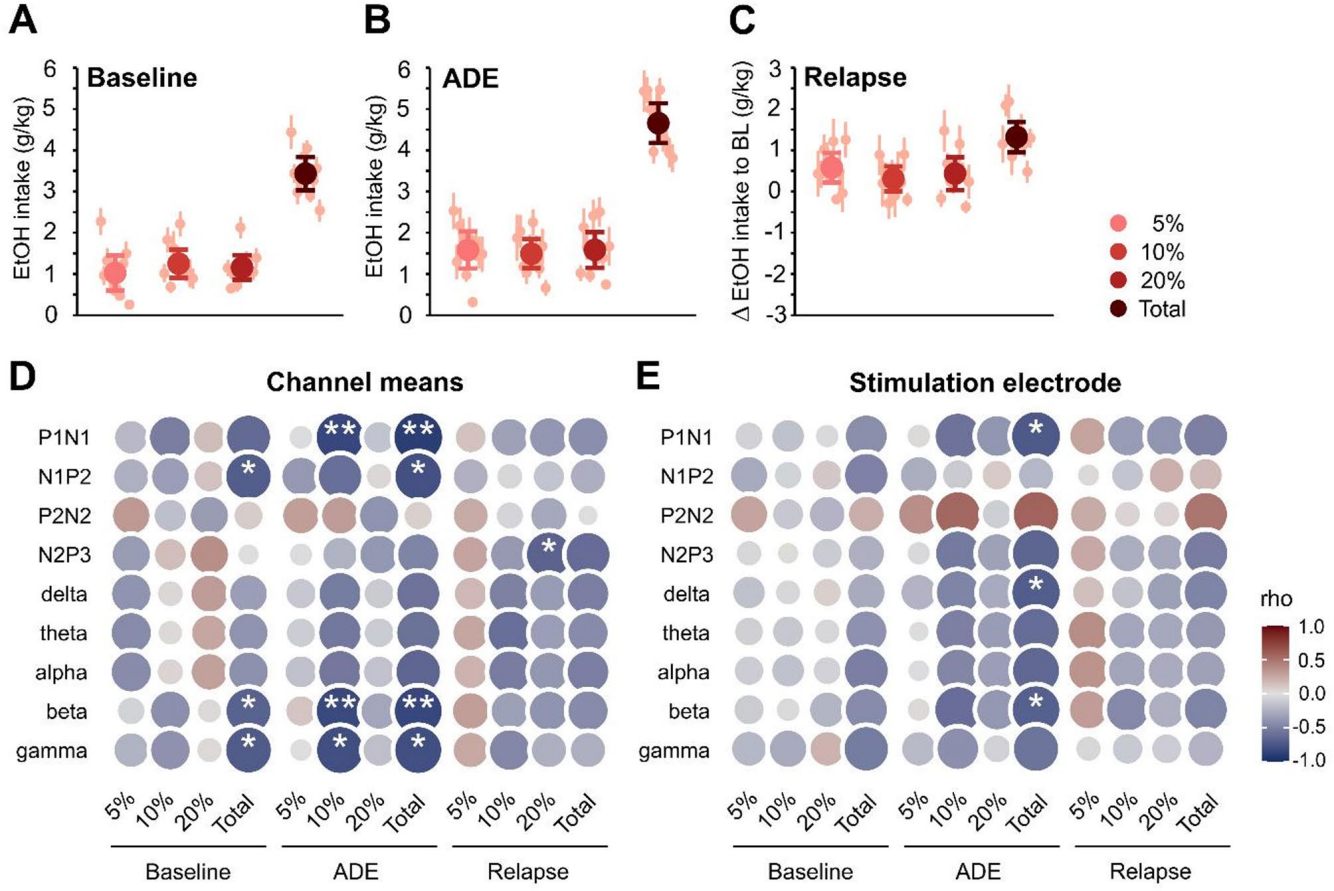
Alcohol consumption and its correlation to post-stimulation event-related activity. The average consumption throughout the experimental timeline of each of the alcohol solutions (5%, 10% and 20% EtOH) and total alcohol intake are presented as the daily means ± SEM of pure EtOH in g/kg. Intake is separated into phases of the paradigm: (**A**) the last week of a drinking phase (baselines, BL), (**B**) the first day following periods of abstinence (alcohol deprivation effect, ADE), and (**C**) relapse (the difference between ADE and the previous BL). Partial Spearman correlation of ERP amplitudes and ERO activity (as event-related spectral perturbation, ERSP) with alcohol consumption following prefrontocortical stimulation as (**D**) means across all channels and (**E**) at the frontocentral stimulation electrode site. Data were controlled for neural activity prior to stimulation. Rho: Spearman’s correlation coefficient with rho ≥ 0.1 = weak, ≥ 0.4 = moderate, ≥ 0.7 = strong, and ≥ 0.9 = very strong correlation. Positive correlations are given in red, negative correlations in blue, with circle sizes increasing with |rho|. Asterisks indicate significant results with *p < 0.05, and **p < 0.01.

Finally, we asked wether electrical stimulation-induced neural enhancement is related to individual past alcohol consumption patterns monitored throughout the ADE paradigm. The mean baseline (BL) total alcohol intake (g/kg/day ± SD) was 3.43 ± 0.57 with rats generally not distinguishing between low, medium and high alcohol content solutions (1.02 ± 0.59 of 5%, 1.25 ± 0.48 of 10% and 1.16 ± 0.42 of 20% alcohol solution, n.s.) (Fig. 5A, Supplementary data 23). Following renewed access to alcohol after deprivation, the animals presented increased consumption of all alcohol concentrations compared with those in the BL, revealing a pronounced ADE (rmANOVA, main effect of drinking phase BL vs. ADE: *p* =  < 0.001, total alcohol consumption: 4.66 ± 0.67, *p* =  < 0.001; 5%: 1.58 ± 0.63, *p* = 0.011; 10%: 1.49 ± 0.49, *p* = 0.189; 20%: 1.59 ± 0.61, *p* = 0.063; Fig. 5B; Supplementary data 23). T- tests against zero values further emphasized significant relapse behaviour for all alcohol concentrations (total: 1.32 ± 0.51, *p* =  < 0.001; 5%: 0.58 ± 0.50, *p* = 0.005; 10%: 0.31 ± 0.42, *p* = 0.044; 20%: 0.43 ± 0.56, *p* = 0.036; Fig. 5C; Supplementary data 23).

Partial correlation analysis was used to examine the relationships between drinking parameters and poststimulation neuronal activity, controlling for individual neural activity before stimulation. However, channel-averaged ERP amplitudes and bandpowers before and after electrical stimulation were weak and nonsignificant except for a negative correlation of N2P3 amplitudes in controls (*ρ* =− 0.73; *p* = 0.017) (Supplementary data 24).

Channel-averaged poststimulation neural activity parameters correlated predominantly negatively with alcohol consumption (Fig. 5D). Moderate to strong effect sizes were observed, particularly for BL consumption of 5% alcohol and total alcohol intake, with significant differences in N1P2 amplitudes (*ρ* =—0.70; *p* = 0.036), betapower (*ρ* =− 0.67, *p* = 0.048) and gammapower (*ρ* =− 0.72, *p* = 0.030) (Fig. 5D, Supplementary data 25). Consumption patterns following alcohol reinstatement (ADE) displayed the most pronounced effects with strong, significant negative correlations between total alcohol intake and P1N1 amplitudes (*ρ* =− 0.89; *p* = 0.001), N1P2 amplitudes (*ρ* =− 0.77; *p* = 0.015), betapower (*ρ* =− 0.83, *p* = 0.005) and gammapower (*ρ* =− 0.78, *p* = 0.012) driven by the consumption of 10% alcohol (P1N1 amplitudes (*ρ* =− 0.84; *p* = 0.004), betapower (*ρ* =− 0.82, *p* = 0.007) and gammapower (*ρ* =− 0.78, *p* = 0.012)). Finally, relapse intensities to higher alcohol solutions correlated with a weaker response to stimulation, with a strong correlation in N2P3 amplitudes to 20% alcohol (*ρ* =− 0.67; *p* = 0.049, Fig. 5D, Supplementary data 25).

Owing to our observation of spatially confined stimulation-related alterations in neural activity in alcohol-dependent rats, a similar partial correlation analysis was performed specifically for the stimulation electrode site (Fig. 5E). The correlations between pre- and poststimulation ERP amplitudes and ERO power were weak and nonsignificant (Supplementary data 24). The overall pattern of correlations was similar to that of channel means, with strong and significant negative correlations between total ADE and P1N1 amplitudes (*ρ* =− 0.73; *p* = 0.026), deltapower (*ρ* =− 0.70, *p* = 0.035) and betapower (*ρ* =—0.68, *p* = 0.043; Fig. 5E, Supplementary data 25).

Together, these results demonstrate that the effectiveness of electrical brain stimulation in remedying chronic alcohol-related cognitive impairments, as shown by normalized event-related neural activity, is mitigated by previous high alcohol consumption, a stronger ADE and higher relapse intensity.

## Discussion

Here, we assessed the capacity of direct cortical stimulation to counteract impairments in event-related ECoG signatures as an index of prefrontal dysfunction following long-term alcohol consumption.

Alcohol-dependent animals exhibit reductions in P1N1 and N1P2 amplitudes, as well as a decline in oscillatory activity across the delta, theta, alpha, and beta frequency bands, whereas gamma power is increased. Moreover, long-term alcohol intake shifted beta activity toward higher frequencies. The observed N1 reduction, along with deficient low-frequency and heightened high-frequency oscillatory activity, has been associated with diminished top-down processing, leading to disturbed executive control and behavioral inhibition^27,55^. Similar neural activity patterns have also been identified in individuals vulnerable to excessive alcohol use and relapse, as well as in first-degree relatives of AUD patients and therefore not only serve as biomarkers to identify those at risk, but also represent targets for therapeutic interventions^56–60^. By applying a single session of electrical stimulation to the prefrontal cortex, we successfully recovered most of these impairments in neural activity in alcohol-dependent rats. Single stimulation sessions have been shown to be effective in supporting short-lived reductions in craving and abstinence in AUD patients, whereas sustained behavioral outcomes require multiple sessions^61–63^, with yet unknown consequences for the level of neuronal activity.

In addition to increased ERP amplitudes and strengthened low-frequency ERO powers, stimulation effectively restored low-beta activity to a level comparable to that of healthy animals. Beta oscillations constitute one of the most reliable AUD endophenotypes^60–64^ and are therefore the strongest indicator of the effectiveness of our neuromodulation approach.

Although we observed a distinct neuroenhancing effect in both healthy controls and alcohol-dependent animals, the stimulation-induced effects in the ADE rat model were weaker and spatially restricted to the stimulation electrode site. Anodal stimulation, as applied here, has been demonstrated to particularly excite neurons with an orthogonal orientation toward the stimulation electrode associated with a horizontal spread of neural activation^48,51^. Indeed, in controls, anodal stimulation effectively enhanced neural activity across a wide area of the frontal lobe. The limited spreading of stimulation-induced neuroenhancement in alcohol-dependent animals suggests impaired prefrontal connectivity, as supported by our nCREANN results, which confirmed reduced linear and non-linear neural transmission. Recent findings indicate that deficient functional connectivity in AUD patients is particularly evident in the beta frequency range^65^. Here, we further demonstrate that, particularly, linear connectivity reduction is most pronounced in the lower beta frequencies, underscoring the relevance of beta subranges in evaluating AUD pathology and the efficacy of neuromodulatory interventions.

We recently demonstrated that our neuroprosthesis does not elicit significant adverse effects or inflammatory responses^52^. Therefore, the neural activity patterns observed in alcohol-dependent animals before and after stimulation may be attributed to alcohol-induced neuroadaptation. Prolonged alcohol intake is known to induce oxidative stress, inflammation and mitochondrial impairment, leading to neural dysfunction and apoptosis^66^, as well as disrupted synaptic connectivity by altering dendritic spine formation and morphology, thereby impairing excitatory signal transmission^67–69^. Alcohol-induced myelin degeneration^70,71^ and resulting white matter atrophy^72^ are particularly relevant for neuromodulatory interventions, as effective and widespread network modulation relies on stimulating myelinated white matter structures^49,73–75^.

Chronic alcohol consumption disrupts glutamatergic projection neurons, the primary excitatory cells that mediate long-range cortical communication. This disruption involves the downregulation of KCa2, KV7 and GIRK ion channels, which increases neuronal excitability and abnormal cell firing^76^, leading to excessive glutamate release and altered glutamate receptor and transporter function^77–79^. Elevated extracellular glutamate is associated with reduced auditory ERP amplitudes^80,81^; decreased delta, theta and alpha power, and increased gamma activity^82^, which is consistent with our observations in alcohol-dependent rats. These chronic alcohol-induced impairments in electrophysiological brain activity and glutamate metabolism reciprocally sustain each other, contributing to the maintenance of addictive behaviors^78,83^. For example, targeting the downregulated metabotropic glutamate receptor type 2 (mGluR2) in alcohol-dependent subjects with the mGluR2 agonist LY379268 reduced alcohol-seeking and relapse behavior^84–86^ while also counteracting the aforementioned disturbances in electrophysiological brain activity^38^.

Glutamatergic hyperexcitation is further exacerbated by diminished expression of gamma-aminobutyric acid (GABA), the primary inhibitory neurotransmitter and counterpart of glutamate. Chronic alcohol exposure disrupts the intrinsic excitability of GABAergic interneurons. The subsequent reduction in spontaneous inhibitory postsynaptic currents and decreased charge transfer are attributed to persistent dysregulation of GABA_A_ receptors^87^. Notably, the GABA_A_ receptor gene GABRA2 is strongly linked to mid-beta oscillatory activity in individuals with alcohol dependence^88^. Beta oscillations are promoted particularly by somatostatin-expressing GABAergic interneurons, whereas parvalbumin (PV)-expressing interneurons suppress beta power, suggesting that the increased high-beta activity observed in alcohol-dependent subjects, including our ADE model, might be based on somatostatin-interneuron hyperactivity and/or PV interneuron hypoactivity induced by prolonged alcohol consumption^89^. PV interneurons, comprising the majority of myelinated cortical inhibitory neurons, are particularly susceptible to alcohol-induced myelin degeneration. This susceptibility likely underlies the disrupted excitatory/inhibitory connectivity and impaired feedforward inhibition^90^ observed in the alcohol-dependent brain.

Finally, the effects of brain surface stimulation depend not only on parameters such as current strength and frequency, but also on the neuron’s prestimulation state. High spontaneous activity before stimulation, combined with low current strengths, as applied in our study, has been shown to induce the inhibition of cell responses^91^. Thus, the hyperaroused neural state characterized by increased high-beta and gamma activity in alcohol-dependent individuals^56,92,93^ might explain the shift in maximum oscillatory activity from high to lower frequency ranges following stimulation and the limited signal spread in our alcohol-dependent animals. Kharas et al.^16^ demonstrated that higher neuronal firing rates during wakefulness restricted signal propagation, whereas at rest, light-induced neural activity robustly spread to adjacent layers. This inverse relationship between arousal and neural coupling supports our finding that hyperarousal impairs the spread of signals. Furthermore, the negative correlation between poststimulation neural activity and alcohol intake suggests reduced stimulation efficacy with increasing alcohol consumption.

In conclusion, we demonstrate the use of multifunctional neuroprosthetics to monitor neuroelectrical signatures associated with chronic alcohol-related cognitive impairments and their rehabilitation through direct electrical stimulation of the PFC. Further studies should examine behavioral outcomes and the impact of our treatment on the reward system in both sexes and different age groups, as well as electrode longevity, to advance the translational potential of this technology. In addition, the evaluation of the duration and stability of stimulation-related effects across single or repeated sessions with interspersed, untreated recordings and/or a between-subject approach are essential to address potential order effects, which cannot be excluded from our design and establish stimulation regimens for clinical application. Finally, the spatially limited efficacy of brain stimulation, underscores the need for further investigations into state-dependent cell type and local neural network properties to elucidate electrical signal transmission in the alcohol-dependent brain to pave the way for disease-specific and personalized neuromodulatory treatments.

## Materials and methods

### Animals

Investigations were performed in Wistar wild-type rats from the Central Institute of Mental Health (CIMH) breeding colony, Mannheim. Only males were used due to ethical considerations, efforts to reduce animal numbers, and technical limitations, as the neuroprosthetic device was optimized for male brain anatomy. The investigated neural mechanisms are fundamental and are not expected to differ significantly by sex.

The rats were housed in single cages (Makrolon®, Type III, Tecniplast Deutschland GmbH, Hohenpeißenberg, Germany) on sawdust bedding (Ssniff—Bedding 3/4 S, Altrogge, Lage, Germany) with Bed-r’Nest material (Datesand Ltd., Bredbury, UK). Pelleted food (V1534-300, ssniff Spezialdiäten GmbH, Soest, Germany) and water were available ad libitum. Housing rooms were temperature (20—22 °C) and humidity (40—55%) controlled with a 12-h light–dark cycle (lights on at 6.00 am).

### Long-term alcohol consumption with repeated deprivation periods

Animals aged 7 weeks were initially habituated to the housing room for 2 weeks. Because simultaneous access to multiple alcohol concentrations has been shown to amplify and prolong the ADE^36,94^, ethanol (VWR International GmbH, Darmstadt, Germany) was then provided ad libitum in 5%, 10%, and 20% (v/v) solutions, in addition to tap water. Bottle positions were changed weekly. After 8 weeks of continuous alcohol availability, the alcohol bottles were removed from the cages and reintroduced after 2 weeks of deprivation. The phases of free access to alcohol and abstinence subsequently alternated randomly with variable durations of drinking between 4–6 weeks and deprivation lasting 2–3 weeks. The long-term alcohol exposure continued over a total duration of 13 months. The alcohol solutions were finally removed, and within the next 2 weeks, the animals underwent daily habituation to the stimulation/recording setup and underwent surgery to implant the neuroprosthetic devices (Fig. 1).

### Manufacturing and implantation of neuroprosthetic interfaces

The fabrication and implantation methods have been detailed in previous works^52,95,96^. In summary, the devices were manufactured via the 3D bioprinter 3DDiscovery™ Evolution (regenHU Ltd., Villaz-St-Pierre, Switzerland) in an additive manufacturing process. The implants comprised a soft silicone base layer (DOWSIL™SE1700, Dow Inc., Midland, USA), a conductive platinum ink layer to form electric circuits (chemPUR, Karlsruhe, Germany) and isolation layers (SYLGARD^TM184^/DOWSIL™ SE734, Dow Inc., Midland, USA). The electrodes were arranged in a 3 × 3 matrix with a distance of 1.5 mm between adjacent electrodes in the mediolateral direction and 2.0 mm in the rostrocaudal direction (Fig. 1B). While eight electrodes (0.2 × 0.2 mm^2^) were dedicated to neural recording, the larger frontocentral electrode (1 × 1 mm^2^) served for recording and bilateral electrical stimulation of the medial PFC at 3.2 mm anterior to bregma. Interconnects were linked to stainless steel microwires (Ø 0.23 mm, 7SS-2T, Science Products GmbH, Hofheim, Germany), and soldered to a 10-pin plug-in connector (BKL 10120653, BKL-Electronic Kreimendahl GmbH). A microwire was attached to the remaining tenth pin, and the skull with a microscrew served as a reference electrode.

Stereotactic implantation was performed under anesthesia with fentanyl (0.005 mg/kg, Hameln Pharma Plus GmbH, Hameln), midazolam (2 mg/kg, Ratiopharm), and medetomidine hydrochloride (0.135 mg/kg, Orion Pharma), which were administered subcutaneously (s.c.) into a nuchal fold. After the skullcap and cranial sutures were exposed, two holes were drilled (Ø 1 mm, H141 205 010, West One Dental) into the skull for the reference screw and to enhance fixation. The skull was trepanned (Ø 6.0 mm, 330205486001060, Meisinger, rpm < 1500) at a position 2.6–3.2 mm to bregma. The implant was placed epidurally on the cortex, with the frontal electrode row located 3.2 mm anterior to bregma, and the reference cable was secured. A silicone elastomer (1A:3B, 3 − 4680, Dow Inc.) was applied to fill the hole. The implant was fixed to the skull using dental cement (Paladur, Kulzer GmbH), and the wound was sutured. Anesthesia was antagonized via s.c. naloxone hydrochloride (0.12 mg/kg, Inresa Arzneimittel GmbH), flumazenil (0.2 mg/kg), and atipamezole hydrochloride (0.75 mg/kg, Orion Pharma). All animals received preventive pain medication (1 mg/kg meloxicam, s.c., Boehringer Ingelheim Vetmedica GmbH) immediately after surgery and on the following day.

### ECoG recording and acute electrical modulation of neural activity

ECoG recordings were conducted more than two weeks after the last alcohol exposure to ensure that animals were free from acute intoxication and acute withdrawal. Recordings were performed on awake animals, initially three days after surgery, and another three days later, immediately following a biphasic, charge-imbalanced electrical pulse stimulation (100 µA/− 80 µA, 130 Hz, 100 µs pulse width; STG4004, MultiChannelSystems) delivered epidurally to the medial PFC through the frontocentral electrode for 20 min as previously described^52^. During stimulation, the animal was placed in a 50 × 50 × 50 cm^3^ Plexiglas box and then directly transferred to the recording setup. Recordings were conducted within an electrically shielded and sound-insulated audiometry booth equipped with a stereo loudspeaker at a distance of 40 cm and an angle of 45° centrally above a rodent sling (Lomir Biomedical Inc.) in which individual animals were placed to reduce movement artefacts. Recordings were performed at a sampling rate of 3 kHz using the Intan RHD2000 USB interface system and recording controller software (Version 1.5.3) with the RHD2132 amplifier chip (Intan Technologies), which was linked to the implant plug-in module via a nanostrip connector (NPD-18-WD-18.0-C-GS, Omnetics Connector Corps.). Sound stimuli to induce auditory event-related neural activity have been generated via the Psychophysics Toolbox (version 3) in MATLAB (version R2019b) and are composed of random series of frequent (standards: 50 ms, 1 kHz, 70 dB sound pressure level (SPL), 87% of trials) and rare (deviants: 50 ms, 2 kHz, 80 dB SPL, 13% of trials) sinusoidal tones with 5-ms onset/offset ramps. Sound sequences were presented in 6 blocks of 5 min with a 1 s interstimulus interval and with deviants interspersed with at least one standard tone.

## Analysis of event-related neural activity

### Data processing

Data processing was performed via the EEGLAB toolbox^97^ (version 2019.1) in MATLAB. Following offline filtering using a 0.1–45 Hz bandpass finite impulse response (FIR) filter (Kaiser window, β = 5.65, filter length 54,330 points), the data were segmented into epochs spanning 100–700 ms relative to stimulus onset for both standard and deviant sounds. Baseline correction was applied using the prestimulus interval between − 100 ms and 0 ms. Identification and exclusion of artifacts and noisy channels were based on a delta criterion of 500 µV and visual inspection before averaging epochs for individual subjects and across all animals (grand average). ERP peak latencies were detected within the following time intervals confirmed by visual inspection: P1: 20–80 ms, N1: 60–150 ms, P2: 70–250 ms, N2: 100–330 ms, P3: 130–600 ms. The amplitudes of the ERP components were calculated as peak-to-peak amplitudes (P1N1, N1P2, P2N2, N2P3) (Fig. 1D).

Event-related oscillatory (ERO) activity in the delta (1–4 Hz), theta (4–8 Hz), alpha (8–12 Hz), beta (12–30 Hz) and gamma (30–45 Hz) frequency bands (Fig. 1D) was determined using the pop_newtimef.m function in EEGLAB on the basis of a fast Fourier transform with 400 datapoints and a padratio of 64. The resulting event-related spectral perturbation (ERSP) was calculated in decibels (dB) (≙ 10*log_10_ (µV^2^/Hz)).

### Statistics

Statistical analyses were carried out in R (version 4.2.3)^98^. For a more robust analysis and greater validity, we imputed missing data for previously excluded channels by applying a machine-learning-based iterative imputation approach using a random forest algorithm (missForest), which has previously demonstrated high reliability across various mixed-type datasets^99^.

ERPs (peak latencies, amplitudes) and EROs (latency and frequency of maximum ERSP within each frequency band) of control animals and long-term alcohol consumers prior to and after brain stimulation were examined by applying multivariate (MANOVA)^100^ and univariate analysis of variance (ANOVA)^101^ with within-factors treatment (pre- vs. poststimulation) and channel location, and between-factor group (controls vs. alcohol-dependent animals), followed by channelwise paired t-tests between treatments for a more detailed extraction of treatment effects. Multiple post-hoc comparisons are reported for detailed analyses of the potential impact of electrode position, along with false discovery rate (FDR)-adjusted* p*-values. For analysis of drinking behaviour, we first calculated the average daily alcohol intake (g/kg bodyweight) for each rat during the last week of each drinking phase (= baseline consumption, BL), on the first day following periods of abstinence (= alcohol deprivation effect (ADE)) and relapse intensities (difference in ADE and previous BLs). Then, a rmANOVA with the factors drinking phase (BL, ADE) and alcohol concentration (5%, 10%, 20%) was conducted to reveal relapse-like drinking following abstinence compared with baseline consumption. Relapse behaviour was statistically tested by conducting t-tests of relapse intensities to zero. Finally, to explore the potential impact of individual alcohol consumption patterns on the efficacy of stimulation, we correlated neural parameters with baseline alcohol consumption, drinking after abstinence (ADE) and relapse intensities. These analyses were conducted using partial Spearman correlations to isolate stimulation-related neural activity while accounting for neural activity prior to stimulation.

## Analysis of functional brain connectivity

### Data preprocessing

To examine how efficiently the applied electrical pulses spread across the prefrontal cortex, we evaluated the connectivity patterns among channels via the non-linear causal relationship estimation by artificial neural network (nCREANN) method^102–105^. For consistent analysis, only rats with available data from a minimum of 8 electrodes were selected in both the control and alcohol groups, resulting in six rats per group. One rat in the control group and three rats in the alcohol group needed interpolation of the missing ninth electrode using EEGLAB.

### Directed functional connectivity

The nCREANN method uses an artificial neural network (ANN) to assess directed connectivity across multiple brain regions using a non-linear multivariate autoregressive (nMVAR) model. This structure represents temporal causality, where the cause influences future outcomes. An MVAR model is a statistical framework used to analyze and predict the behavior of multiple time series variables (multiple brain regions’ activities captured by the electrodes) that may influence each other over time. In a classical MVAR model, the current values of multiple variables are expressed as linear combinations of their past values. This model captures the linear dependencies between time series data from different variables.

Considering that complex non-linear behaviors of the nervous system have been observed at all levels^106^, from a single neuron to the system, linear approaches may oversimplify the complicated dynamics of brain function. It has been shown that non-linear interactions play crucial roles in structuring information flow between cortical areas^107,108^. Several lines of evidence point to the usefulness of both linear and non-linear concepts in enhancing our understanding of neurodynamics at macroscale levels^109–113^.

The non-linear MVAR model generalizes the MVAR model by incorporating non-linear functions to describe the relationships between the current and past values of the variables. That is, the current state of each variable is not just a simple linear combination of its past states and those of other variables, but can also include more complex, non-linear interactions.

For a given time series ${\mathbf{x}}\left( n \right) \in {\mathbb{R}}^{M}$ of length L, a non-linear MVAR model of order $p$ is defined as

1 $${\mathbf{x}}\left( n \right) = {\varvec{f}}\left( {{\mathbf{x}}_{p} } \right) + {{\varvec{\upsigma}}}\left( n \right)$$

The vector ${\mathbf{x}}_{p}={\left[{x}_{1}\left(n-1\right),{x}_{2}\left(n-1\right), \cdots ,{x}_{M}\left(n-p\right) \right]}^{\text{T}}$ represents $p$ past samples of (M) multivariate time series. The noise vector, ${\varvec{\upsigma}}\left(n\right)= {\left[{\sigma }_{1}, {\sigma }_{2}, \dots , {\sigma }_{M} \right]}^{T},$ denotes the model residual, and the non-linear function ${\varvec{f}}\left(.\right)$ quantitatively describes how the $p$ previous samples influence the future values. In the nCREANN method, the function ${\varvec{f}}$ is separated into linear and non-linear components

2 $${\varvec{f}} = {\varvec{f}}^{Lin} + {\varvec{f}}^{NonLin} .$$

The linear connectivity $({lC}_{i\to j})$ describes the linear impact of the *i*th region on the *j*th region on the basis of the ${{\varvec{f}}}^{Lin}$ part. Furthermore, by using the information contained of ${{\varvec{f}}}^{NonLin}$, the Non-linear Connectivity ${(NC}_{i\to j})$ is deduced to determine the degree of the non-linear causal influence of ${x}_{i}$ on ${x}_{j}$.

In the present study, nCREANN was applied to the ECoG signals of the nine electrodes in the alcohol and control groups. The data points of the trials in the time interval from − 100–700 ms after stimulus onset were considered for the connectivity analysis. To create data of sufficient length for training the network, we concatenated the individual trials. The single trial ‘contrast’ signals, ${Contrast}_{single}$, were generated by subtracting the average of trials of the ‘standard’ condition from every single trial of the ‘deviant’ condition, $Deviant_{single}$:

3 $$Contrast_{single} = Deviant_{single} - \frac{1}{N}\mathop \sum \limits_{n = 1}^{N} Standard_{single}^{n}$$

Where N is the total number of trials in the ‘standard’ condition.

The proper model order for the nMVAR was determined on the basis of the Akaike and Schwartz criteria^114^. The model order $p=8$ was considered the same for all the subjects in both groups.

A multilayer perceptron neural network with one hidden layer and 10 hidden neurons was trained. During training, the network attempts to predict $\mathbf{x}\left(n\right)$ at its output based on the input values, ${\mathbf{x}}_{p}$. The gradient descent error back-propagation (EBP) with momentum (α) and adaptive learning rate (η) served as the training algorithm. To ensure generalizability, the early stopping technique was applied. The data were split into 80% training, 10% validation, and 10% testing sets for each fold in the tenfold permuted cross-validation approach. The network parameters are updated every time an input is presented (the ‘incremental’ training mode).

The validity of the nMVAR model was evaluated on the basis of the mean square error (MSE) and R-squared values of the training and test data. The MSE is one of the most commonly used statistics to evaluate the performance of a network. R-squared (R^2^), or the coefficient of determination, is a statistical metric used to assess how well a model fits and to evaluate its goodness of fit.

A properly trained network exhibits not only a small training error but also a test error within the range of the training error. Furthermore, comparable R^2^ values for the training and test sets highlight the network’s suitable generalizability. Moreover, we assessed the significance of the connectivity values using a randomisation test with the generation of 100 datasets via the time-shifted surrogate technique^115^. Without altering the dynamics of any time series, this technique eliminates any causal relationship between the signals. The network configurations were the same as those used for the original data when nCREANN was used on the surrogate data.

### Statistics

Since the linear and non-linear connectivity values were not normally distributed (as assessed by the Kolmogorov–Smirnov test), a nonparametric Mann–Whitney U statistic test was conducted, and the effect size was computed.

## Supplementary Information

Below is the link to the electronic supplementary material.

Supplementary Material 1

Supplementary Material 2

Supplementary Material 3

Supplementary Material 4

Supplementary Material 5

Supplementary Material 6

Supplementary Material 7

Supplementary Material 8

Supplementary Material 9

Supplementary Material 10

Supplementary Material 11

Supplementary Material 12

Supplementary Material 13

Supplementary Material 14

Supplementary Material 15

Supplementary Material 16

Supplementary Material 17

Supplementary Material 18

Supplementary Material 19

Supplementary Material 20

Supplementary Material 21

Supplementary Material 22

Supplementary Material 23

Supplementary Material 24

Supplementary Material 25

## Data Availability

All the data needed to evaluate the conclusions in the paper are presented in the paper and/or the Supplementary Materials. The code can be found on GitHub (https://github.com/habeltb/Multimodal_ECoG_ratPFC_alcoholism).
