# Supplementary material for: Chronic alcohol-induced brain states limit propagation of direct cortical stimulation
Source: Sci Rep. 2025 Oct 10;15:35407. doi: 10.1038/s41598-025-21802-z (PMC12514308; doi:10.1038/s41598-025-21802-z)
Supplement: Supplementary file 18 — Supplementary Material 18 [file 41598_2025_21802_MOESM18_ESM.pdf]

Table S1 Mann–Whitney U statistics comparing linear prefrontal connectivity between healthy controls and alcohol-addicted rats. A) Significant *p*-values given in bold, italic. **B)** Effect size (rank-biseral correlation) with $|r| \geq 0.1$ = small, $|r| \geq 0.3$ = medium and $|r| \geq 0.5$ = large effects (given in bold, italic).

A

To	From →	PR	MR	FR	FC	PC	MC	FL	ML	PL
	PR		0.065	0.180	0.065	0.132	0.065	0.093	***0.041***	0.310
	MR	***0.015***		0.065	0.132	0.093	0.485	0.699	0.180	0.589
	FR	***0.015***	***0.026***		0.132	0.394	***0.026***	***0.041***	0.065	0.177
	FC	0.310	0.180	0.132		0.093	0.093	0.310	0.240	0.485
	PC	***0.015***	***0.015***	0.132	***0.002***		***0.041***	0.429	0.485	0.310
	MC	***0.015***	0.180	***0.015***	***0.041***	0.132		0.132	0.065	0.180
	FL	0.394	0.485	0.093	0.093	0.485	0.180		0.065	0.589
	ML	0.310	0.065	0.132	***0.026***	0.310	0.065	***0.026***		0.126
	PL	***0.015***	0.310	0.589	0.485	0.394	0.240	0.240	0.240	

B

To	From →	PR	MR	FR	FC	PC	MC	FL	ML	PL
	PR		***0.555***	0.416	***0.555***	0.462	***0.555***	***0.508***	***0.601***	0.324
	MR	***0.693***		***0.555***	0.462	***0.508***	0.231	0.139	0.416	0.185
	FR	***0.693***	***0.647***		0.462	0.277	***0.647***	***0.601***	***0.555***	0.440
	FC	0.324	0.416	0.462		***0.508***	***0.508***	0.324	0.370	0.231
	PC	***0.693***	***0.693***	0.462	***0.832***		***0.601***	0.275	0.231	0.324
	MC	***0.693***	0.416	***0.693***	***0.601***	0.462		0.462	***0.555***	0.416
	FL	0.277	0.231	***0.508***	***0.508***	0.231	0.416		***0.555***	0.185
	ML	0.324	***0.555***	0.462	***0.647***	0.324	***0.555***	***0.647***		0.495
	PL	***0.693***	0.324	0.185	0.231	0.277	0.370	0.370	0.370	

Electrode sites are labelled according to their position above the medial PFC as frontocentral (FC), frontal left (FL), frontal right (FR), medial central (MC), medial left (ML), medial right (MR), posterior central (PC), posterior left (PL) and posterior right (PR).

Table S2 Mann–Whitney U statistics comparing non-linear prefrontal connectivity between healthy controls and alcohol-addicted rats. A) Significant *p*-values given in bold, italic. **B)** Effect size (rank-biseral correlation) with $|r| \geq 0.1$ = small, $|r| \geq 0.3$ = medium and $|r| \geq 0.5$ = large effects.

A

To		From								
		PR	MR	FR	FC	PC	MC	FL	ML	PL
	PR		0.699	***0.041***	0.132	***0.004***	***0.002***	0.132	0.114	0.065
	MR	0.132		0.132	0.132	0.132	0.699	0.240	0.065	0.132
	FR	0.485	0.310		0.177	0.180	0.132	0.240	0.180	0.485
	FC	0.180	0.429	0.065		0.310	0.132	0.082	0.394	0.394
	PC	0.132	0.240	0.082	0.937		***0.026***	0.180	0.180	0.082
	MC	0.240	0.537	0.329	0.329	***0.026***		0.329	0.177	0.537
	FL	0.818	0.548	0.310	0.310	0.126	0.537		0.310	0.589
	ML	0.180	0.485	0.329	0.247	0.937	0.240	0.247		0.310
	PL	0.310	0.429	0.589	0.240	0.132	0.699	0.132	0.818	

B

To		From								
		PR	MR	FR	FC	PC	MC	FL	ML	PL
	PR		0.139	***0.601***	0.462	***0.786***	***0.832***	0.462	***0.539***	***0.555***
	MR	0.462		0.462	0.462	0.462	0.139	0.370	***0.555***	0.462
	FR	0.231	0.324		0.440	0.416	0.462	0.370	0.416	0.231
	FC	0.416	0.275	***0.555***		0.324	0.462	***0.550***	0.277	0.277
	PC	0.462	0.370	***0.550***	0.046		***0.647***	0.416	0.416	***0.550***
	MC	0.370	0.220	0.330	0.330	***0.647***		0.330	0.440	0.220
	FL	0.092	0.231	0.324	0.324	0.495	0.220		0.324	0.185
	ML	0.416	0.231	0.330	0.385	0.046	0.370	0.385		0.324
	PL	0.324	0.275	0.185	0.370	0.462	0.139	0.462	0.092	

Electrode sites are labelled according to their position above the medial PFC as frontocentral (FC), frontal left (FL), frontal right (FR), medial central (MC), medial left (ML), medial right (MR), posterior central (PC), posterior left (PL) and posterior right (PR).
