# Supplementary material for: Chronic alcohol-induced brain states limit propagation of direct cortical stimulation
Source: Sci Rep. 2025 Oct 10;15:35407. doi: 10.1038/s41598-025-21802-z (PMC12514308; doi:10.1038/s41598-025-21802-z)
Supplement: Supplementary file 21 — Supplementary Material 21 [file 41598_2025_21802_MOESM21_ESM.pdf]

Table S3 Mann–Whitney U statistics comparing linear prefrontal connectivity at low-beta frequencies (12 Hz – 15 Hz) between healthy controls and alcohol-addicted rats. A) Significant *p*-values given in bold, italic. **B)** Effect size (rank-biseral correlation) with |*r*| ≥ 0.1 = small, |*r*| ≥ 0.3 = medium and |*r*| ≥ 0.5 = large effects (given in bold, italic).

A

		From								
		PR	MR	FR	FC	PC	MC	FL	ML	PL
**To**	PR		0.229	0.400	0.533	0.413	0.071	1.000	0.100	0.533
	MR	0.400		0.400	0.133	0.571	0.057	0.095	0.143	1.000
	FR	0.533	0.343		0.629	1.000	0.114	0.114	0.393	0.333
	FC	0.800	0.857	0.571		0.857	0.250	0.857	0.381	0.267
	PC	0.400	0.800	0.800	0.800		0.533	0.400	0.190	0.533
	MC	0.071	0.533	0.250	0.381	0.800		0.381	1.000	0.400
	FL	0.533	***0.036***	0.250	0.533	0.800	0.343		0.413	1.000
	ML	0.400	0.133	0.400	1.000	0.250	0.629	0.250		0.400
	PR	0.700	0.667	1.000	1.000	0.400	0.500	1.000	0.143	

B

		From								
		PR	MR	FR	FC	PC	MC	FL	ML	PL
**To**	PR		***0.535***	0.401	0.378	0.327	***0.685***	0.000	***0.802***	0.378
	MR	0.401		0.401	***0.756***	0.293	***0.802***	***0.732***	***0.580***	0.224
	FR	0.378	0.408		0.267	0.000	***0.612***	***0.668***	0.369	***0.598***
	FC	0.189	0.146	0.264		0.146	0.474	0.146	0.439	***0.567***
	PC	0.401	0.258	0.258	0.189		0.378	***0.632***	***0.586***	0.378
	MC	***0.685***	0.378	0.474	0.439	0.189		0.439	0.000	***0.632***
	FL	0.378	***0.791***	0.474	0.378	0.189	0.408		0.327	0.000
	ML	0.401	***0.756***	***0.516***	0.000	0.474	0.267	0.474		***0.632***
	PR	0.267	0.359	0.224	0.120	***0.632***	***0.671***	0.120	***0.580***	

Electrode sites are labelled according to their position above the medial PFC as frontocentral (FC), frontal left (FL), frontal right (FR), medial central (MC), medial left (ML), medial right (MR), posterior central (PC), posterior left (PL) and posterior right (PR).

Table S4 Mann–Whitney U statistics comparing non-linear prefrontal connectivity at low-beta frequencies (12 Hz – 15 Hz) between healthy controls and alcohol-addicted rats. A) Significant *p*-values given in bold, italic. **B)** Effect size (rank-biseral correlation) with |*r*| ≥ 0.1 = small, |*r*| ≥ 0.3 = medium and |*r*| ≥ 0.5 = large effects (given in bold, italic).

A

		From								
		PR	MR	FR	FC	PC	MC	FL	ML	PL
**To**	PR		0.393	1.000	0.393	1.000	0.393	0.486	0.786	0.393
	MR	0.571		1.000	0.393	0.571	0.730	0.800	0.786	0.700
	FR	0.905	0.857		0.571	0.629	0.905	0.886	1.000	1.000
	FC	0.250	0.786	1.000		0.786	1.000	0.571	0.786	0.857
	PC	0.393	0.786	0.786	0.393		0.400	0.571	0.786	0.400
	MC	0.905	1.000	0.629	0.905	0.629		0.486	0.486	0.533
	FL	0.393	0.400	1.000	0.905	0.786	0.730		0.114	0.730
	ML	0.857	1.000	1.000	0.393	1.000	0.730	0.905		0.786
	PR	1.000	0.100	0.400	0.400	0.857	0.857	0.629	0.686	

B

		From								
		PR	MR	FR	FC	PC	MC	FL	ML	PL
**To**	PR		0.369	0.000	0.369	0.000	0.369	0.306	0.158	0.369
	MR	0.264		0.053	0.369	0.264	0.163	0.189	0.158	0.267
	FR	0.082	0.146		0.264	0.267	0.082	0.102	0.053	0.000
	FC	0.474	0.158	0.000		0.158	0.000	0.264	0.158	0.146
	PC	0.369	0.158	0.158	0.369		0.401	0.264	0.158	0.401
	MC	0.082	0.053	0.267	0.082	0.267		0.306	0.306	0.378
	FL	0.369	0.401	0.053	0.082	0.158	0.163		***0.668***	0.163
	ML	0.146	0.053	0.053	0.369	0.053	0.163	0.082		0.158
	PR	0.000	***0.802***	0.445	0.401	0.134	0.134	0.267	0.204	

Table S5 Mann–Whitney U statistics comparing linear prefrontal connectivity at mid-beta frequencies (15 Hz – 18 Hz) between healthy controls and alcohol-addicted rats. A) Significant *p*-values given in bold, italic. **B)** Effect size (rank-biseral correlation) with |*r*| ≥ 0.1 = small, |*r*| ≥ 0.3 = medium and |*r*| ≥ 0.5 = large effects (given in bold, italic).

A

		From								
		PR	MR	FR	FC	PC	MC	FL	ML	PL
To	PR		0.056	0.730	0.905	0.485	0.931	0.310	0.931	0.690
	MR	0.082		0.421	0.886	0.485	0.548	0.143	0.421	0.699
	FR	0.063	1.000		0.931	0.548	0.548	0.690	1.000	0.177
	FC	0.413	0.730	0.690		1.000	0.286	1.000	0.167	0.067
	PC	0.792	0.730	0.190	0.730		0.052	0.792	0.931	0.662
	MC	0.257	0.222	1.000	0.905	0.931		0.931	1.000	0.537
	FL	0.714	0.111	0.429	0.556	0.610	0.222		0.222	0.792
	ML	0.690	0.222	0.841	0.556	0.556	0.421	0.690		0.818
	PL	0.190	1.000	0.556	0.413	0.857	0.250	0.190	0.286	

B

		From								
		PR	MR	FR	FC	PC	MC	FL	ML	PL
To			***0.628***	0.163	0.082	0.231	0.055	0.363	0.055	0.165
	PR	***0.550***		0.297	0.102	0.231	0.231	***0.580***	0.297	0.139
	MR	***0.653***	0.033		0.055	0.258	0.231	0.165	0.000	0.440
	FR	0.327	0.163	0.165		0.033	0.408	0.053	***0.516***	***0.607***
	FC	0.110	0.163	0.490	0.163		***0.606***	0.110	0.055	0.165
	PC	0.405	0.429	0.000	0.082	0.055		0.055	0.033	0.220
	MC	0.172	***0.572***	0.275	0.245	0.202	0.429		0.429	0.110
	FL	0.165	0.429	0.099	0.245	0.245	0.297	0.165		0.092
	ML	0.490	0.000	0.245	0.327	0.134	0.474	0.490	0.408	

Table S6 Mann–Whitney U statistics comparing non-linear prefrontal connectivity at mid-beta frequencies (15 Hz – 18 Hz) between healthy controls and alcohol-addicted rats. A) Significant *p*-values given in bold, italic. **B)** Effect size (rank-biseral correlation) with $|r| \geq 0.1$ = small, $|r| \geq 0.3$ = medium and $|r| \geq 0.5$ = large effects (given in bold, italic).

A

To		From								
		PR	MR	FR	FC	PC	MC	FL	ML	PL
	PR		0.699	0.537	0.429	0.429	0.730	0.841	0.931	0.905
	MR	1.000		0.792	0.537	1.000	***0.030***	1.000	0.429	0.699
	FR	0.699	0.937		0.841	1.000	0.114	***0.030***	0.537	0.937
	FC	0.485	0.310	0.310		0.792	0.662	0.247	0.690	0.413
	PC	0.310	0.792	0.310	0.126		0.413	0.421	0.690	0.310
	MC	0.931	1.000	0.537	0.699	0.537		1.000	0.429	0.537
	FL	0.662	1.000	0.841	0.931	0.690	0.690		0.476	0.556
	ML	0.485	1.000	1.000	0.792	0.914	0.111	***0.008***		0.841
	PR	0.610	0.662	0.589	0.413	0.841	0.905	1.000	0.730	

B

To		From								
		PR	MR	FR	FC	PC	MC	FL	ML	PL
	PR		0.139	0.220	0.275	0.275	0.163	0.099	0.055	0.082
	MR	0.000		0.110	0.220	0.000	***0.661***	0.000	0.275	0.139
	FR	0.139	0.046		0.099	0.033	***0.539***	***0.661***	0.220	0.046
	FC	0.231	0.324	0.363		0.110	0.165	0.385	0.165	0.327
	PC	0.324	0.110	0.363	0.495		0.327	0.297	0.165	0.363
	MC	0.055	0.000	0.220	0.139	0.220		0.033	0.275	0.220
	FL	0.165	0.000	0.099	0.055	0.165	0.165		0.270	0.245
	ML	0.231	0.000	0.000	0.110	0.067	***0.572***	***0.826***		0.099
	PR	0.202	0.165	0.185	0.327	0.099	0.082	0.033	0.163	

Table S7 Mann–Whitney U statistics comparing linear prefrontal connectivity at high-beta frequencies (18 Hz – 30 Hz) between healthy controls and alcohol-addicted rats. A) Significant *p*-values given in bold, italic. **B)** Effect size (rank-biseral correlation) with |*r*| ≥ 0.1 = small, |*r*| ≥ 0.3 = medium and |*r*| ≥ 0.5 = large effects (given in bold, italic).

A

					**From**				
**To**	PR	MR	FR	FC	PC	MC	FL	ML	PL
PR		1.000	***0.017***	***0.017***	0.093	0.589	0.589	0.132	0.931
MR	0.589		0.052	0.132	0.310	1.000	0.310	0.310	0.931
FR	0.792	0.429		0.537	0.841	0.310	0.818	***0.026***	0.537
FC	0.841	0.931	0.548		0.931	0.699	0.589	0.247	0.429
PC	0.126	0.429	0.662	0.792		0.394	0.699	1.000	0.589
MC	0.699	0.699	***0.009***	0.132	0.132		0.818	0.394	0.421
FL	0.699	0.589	0.052	0.180	0.589	0.394		***0.026***	0.792
ML	0.429	0.699	0.429	***0.026***	0.589	0.240	***0.041***		0.537
PL	1.000	0.589	0.931	0.537	0.662	0.082	0.180	1.000	

B

					**From**				
**To**	PR	MR	FR	FC	PC	MC	FL	ML	PL
PR		0.000	***0.716***	***0.716***	***0.508***	0.185	0.185	0.462	0.055
MR	0.185		***0.606***	0.462	0.324	0.000	0.324	0.324	0.055
FR	0.110	0.275		0.220	0.099	0.324	0.092	***0.647***	0.220
FC	0.099	0.055	0.231		0.055	0.139	0.185	0.385	0.275
PC	0.495	0.275	0.165	0.110		0.277	0.139	0.000	0.185
MC	0.139	0.139	***0.740***	0.462	0.462		0.092	0.277	0.297
FL	0.139	0.185	***0.606***	0.416	0.185	0.277		***0.647***	0.110
ML	0.275	0.139	0.275	***0.647***	0.185	0.370	***0.601***		0.220
PL	0.033	0.185	0.055	0.220	0.165	***0.550***	0.416	0.033	

Table S8 Mann–Whitney U statistics comparing non-linear prefrontal connectivity at high-beta frequencies (18 Hz – 30 Hz) between healthy controls and alcohol-addicted rats. A) Significant *p*-values given in bold, italic. **B)** Effect size (rank-biseral correlation) with |*r*| ≥ 0.1 = small, |*r*| ≥ 0.3 = medium and |*r*| ≥ 0.5 = large effects (given in bold, italic).

A

To		From								
		PR	MR	FR	FC	PC	MC	FL	ML	PL
	PR		1.000	0.095	0.190	0.792	0.662	0.589	0.329	0.329
	MR	0.818		0.662	0.662	0.841	0.240	0.699	0.818	0.818
	FR	0.082	0.247		0.310	0.537	0.937	0.329	1.000	0.421
	FC	0.126	0.247	0.548		0.537	0.394	0.247	0.937	0.421
	PC	0.792	0.537	0.310	0.841		0.180	0.240	0.537	0.792
	MC	0.485	0.818	0.310	0.429	1.000		1.000	0.394	0.247
	FL	0.052	1.000	0.286	0.699	0.914	0.792		0.589	0.792
	ML	0.485	0.818	0.841	0.247	1.000	0.180	0.589		0.329
	PL	0.792	1.000	0.421	0.690	0.151	0.792	0.792	1.000	

B

To		From								
		PR	MR	FR	FC	PC	MC	FL	ML	PL
	PR		0.000	***0.561***	0.490	0.110	0.165	0.185	0.330	0.330
	MR	0.092		0.165	0.165	0.099	0.370	0.139	0.092	0.092
	FR	***0.550***	0.385		0.363	0.220	0.046	0.330	0.000	0.297
	FC	0.495	0.385	0.231		0.220	0.277	0.385	0.046	0.297
	PC	0.110	0.220	0.363	0.099		0.416	0.370	0.220	0.110
	MC	0.231	0.092	0.363	0.275	0.000		0.000	0.277	0.385
	FL	***0.606***	0.033	0.408	0.139	0.067	0.110		0.185	0.110
	ML	0.231	0.092	0.099	0.385	0.033	0.416	0.185		0.330
	PL	0.110	0.000	0.297	0.165	0.495	0.110	0.110	0.000	
